# Delayed diagnosis of severe diabetic ketoacidosis associated with a sodium-glucose cotransporter 2 inhibitor: a case report

**DOI:** 10.1093/omcr/omad074

**Published:** 2023-07-18

**Authors:** Hiroaki Taniguchi, Takero Terayama, Soichiro Seno, Nobuaki Kiriu, Hiroshi Kato, Yasumasa Sekine, Tetsuro Kiyozumi

**Affiliations:** Department of Traumatology and Critical Care Medicine, National Defense Medical College Hospital, Tokorozawa City, Saitama, Japan; Department of Traumatology and Critical Care Medicine, National Defense Medical College Hospital, Tokorozawa City, Saitama, Japan; Department of Traumatology and Critical Care Medicine, National Defense Medical College Hospital, Tokorozawa City, Saitama, Japan; Department of Traumatology and Critical Care Medicine, National Defense Medical College Hospital, Tokorozawa City, Saitama, Japan; Department of Traumatology and Critical Care Medicine, National Defense Medical College Hospital, Tokorozawa City, Saitama, Japan; Department of Traumatology and Critical Care Medicine, National Defense Medical College Hospital, Tokorozawa City, Saitama, Japan; Department of Traumatology and Critical Care Medicine, National Defense Medical College Hospital, Tokorozawa City, Saitama, Japan

## Abstract

Sodium-glucose cotransporter 2 (SGLT2) inhibitors are used to treat patients with type 2 diabetes mellitus but may induce diabetic ketoacidosis (DKA). Owing to their pharmacological mechanisms, they cause a different pathogenesis to that of typical DKA and require special attention in terms of blood glucose concentrations and acidosis. We describe a case of prolonged acidosis because of failure to immediately discover the use of an SGLT2 inhibitor. Compared with typical DKA, SGLT2 inhibitor-associated DKA requires earlier and longer glucose supplementation. SGLT2 inhibitors are specific aetiological factors in DKA, and their use should be suspected when the patient presents with mild hyperglycaemia or prolonged acidosis.

## INTRODUCTION

Sodium-glucose cotransporter 2 (SGLT2) inhibitors are used as treatment for type 2 diabetes mellitus (T2DM) to reduce morbidity and mortality [1]. However, their use is associated with a higher risk of diabetic ketoacidosis (DKA) [2]. Additionally, patients with SGLT2 inhibitor-associated DKA may take longer to recover than a typical case of DKA [3]. Herein, we report a case of SGLT2 inhibitor-associated DKA that worsened during treatment, highlighting the necessity of suspecting SGLT2 inhibitor use when acidosis is prolonged.

**Table 1:** Blood test on admission.

Variables	Results
Arterial blood gas analysis^a^	
pH	6.939
PaCO_2_ (mmHg)	7.3
PaO_2_ (mmHg)	158
HCO_3_ (mmol/L)	4.7
Base excess (mmol/L)	−28.9
Lactate (mmol/L)	1.9
Complete blood cell count	
White blood cell count (×10^3^/μL)	26.9
Neutrophil (%)	89.2
Lymphocyte (%)	7.6
Haemoglobin (g/dL)	12.4
Platelet count (×10^4^/μL)	33.0
Blood biochemistry	
Total bilirubin (mg/dL)	0.19
AST (U/L)	85
ALT (U/L)	40
Creatinine (mg/dL)	1.58
Sodium (mmol/L)	153
Potassium (mmol/L)	7.4
Chlorine (mmol/L)	117
C-reactive protein (mg/dL)	11.5
Blood glucose (mg/dL)	269

AST, aspartate aminotransferase; ALT, alanine aminotransferase.

^a^Blood gas analysis was performed under oxygen administration at 10 L/min using a mask with a reservoir.

## CASE REPORT

**Figure 1:**
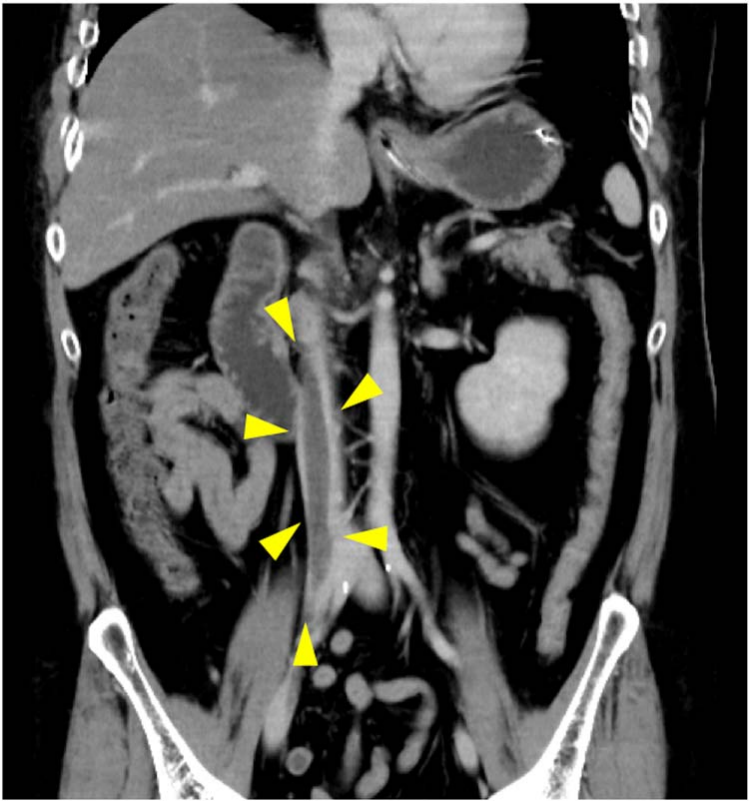
Contrast-enhanced computed tomography (coronal view) on admission. A thrombus (yellow arrowheads) is located in the inferior vena cava from the right iliac vein.

A 40-year-old man was admitted to our hospital with loss of consciousness and hyperventilation. We could not initially obtain the relevant medical history but later discovered that he had been admitted to a local hospital 1 month earlier for hyperglycaemia, diagnosed with T2DM and administered an SGLT2 inhibitor (empagliflozin). On arrival at our hospital, the Glasgow Coma Scale score was 10 (E3V3M4), heart rate was 82 beats/min, radial artery was not palpable and respiratory rate was 20 breaths/min. An infectious lesion was identified in the right inguinal area, likely representing a scar caused by central venous catheter placement. He had severe metabolic acidosis, hyperglycaemia, inflammation and an electrolyte imbalance (Table 1). Urine ketone test results were strongly positive. Computed tomography revealed a thrombus in the inferior vena cava (Fig. 1). He was diagnosed with severe DKA and thrombophlebitis and treated with fluid management, electrolyte management, insulin and antibiotics.

**Figure 2:**
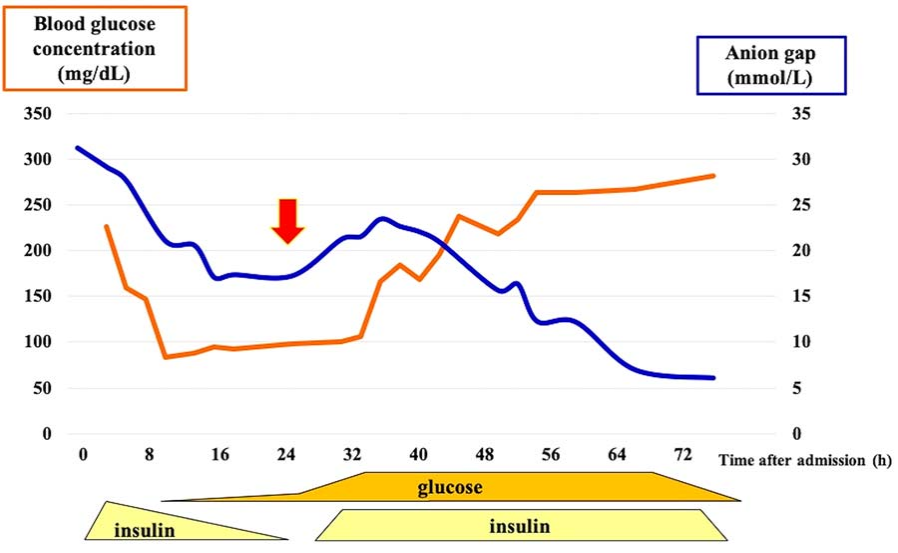
Change in the status of DKA. The patient’s blood glucose concentration quickly decreased from 200 to 100 mg/dL after admission. His anion gap decreased from ~30 to 15 mmol/L. Therefore, the insulin dose was reduced. However, his acidosis worsened, and glucose was gradually administered 24 h after admission (red arrow), which markedly improved the condition.

Figure 2 displays the changes in the anion gap and blood glucose concentration over time, as well as the course of treatment. Ten hours after admission, the blood glucose concentration rapidly decreased to a concentration of 100 mg/dL. Therefore, the insulin dosage was reduced, and its route was switched to subcutaneous administration, even though the acidosis was not completely resolved.

However, acidosis worsened 24 h after admission. At that point, SGLT2 inhibitor involvement was suspected and discovered. Glucose supplementation was initiated, and the dose was gradually increased to 15 g/h. Acidosis progressively improved and finally normalized 60 h after admission. Insulin was continuously administered for blood glucose control until 90 h after admission. The serum ketone body levels at the time of admission were as follows: acetoacetic acid, 3810 (normal range, 13.0–69.0) μmol/L; beta-hydroxybutyric acid, 5590 (normal level, < 76.0) μmol/L; and total ketones, 9400 (normal range, 26.0–122.0) μmol/L. Blood cultures revealed the presence of methicillin-sensitive *Staphylococcus aureus*. Antibiotic treatment was completed, and the patient was discharged 4 weeks after admission.

## DISCUSSION

SGLT2 inhibitors are therapeutic agents for T2DM, targeting SGLT2 and preventing glucose reabsorption from the proximal renal tubules [4]. They reportedly prevent cardiac and renal diseases and reduce mortality in patients with T2DM [1]. However, amongst the reported adverse effects, urinary tract infections are the most common [5]. Additionally, SGLT2 inhibitors increase the risk of DKA, a rare but serious adverse event, 2–3-folds compared with other oral T2DM drugs [2]. In a previous study, the prevalence of DKA amongst patients taking SGLT2 inhibitors was 0.43%; the most common precipitating factors were infection (32.6%), insulin nonadherence (13.7%), pancreatitis (4.7%) and surgery (2.3%) [6]. The reported mortality rate is 1.54% [7], and appropriate treatment is crucial.

The pathophysiology of SGLT2 inhibitor-associated DKA differs from that of typical cases, and caution should be exercised in cases of mild hyperglycaemia and prolonged acidosis. SGLT2 inhibitors decrease blood glucose via urinary excretion. This reduction decreases insulin secretion by the pancreas, resulting in an increased glucagon-to-insulin ratio and ketoacidosis [8]. In contrast, typical DKA results in hyperglycaemia, metabolic acidosis and ketosis owing to decreased insulin concentrations and increased concentrations of catecholamines, cortisol and other counterregulatory hormones. Therefore, in terms of the clinical course, the blood glucose concentration and duration of acidosis differ. SGLT2 inhibitor-associated DKA reportedly yields a lower blood glucose concentration (229 vs. 458.5 mg/dL), longer time to DKA recovery (39 vs. 19 h) and longer insulin administration (41 vs. 21 h) than typical DKA [3, 6]. Characteristically, SGLT2 inhibitors can cause euglycaemic DKA, marked by a pH <  7.3, HCO_3_ concentration < 18 mEq/L, ketosis and blood glucose concentration < 200 mg/dL, which may lead to a delay in appropriate treatment [9]. The use of SGLT2 inhibitors should be suspected when patients with DKA exhibit mild hyperglycaemia or prolonged acidosis. In general, DKA treatment involves the correction of fluid and electrolyte balance and insulin administration. In addition, euglycaemic DKA may require glucose administration to prevent hypoglycaemia [10], and even more so in SGLT2 inhibitor-associated DKA, considering its pharmacological mechanism [11].

In this case, DKA was likely caused by *S. aureus* bacteraemia and a thrombus associated with a central venous catheter at the previous hospital. At the time of DKA diagnosis, insufficient information regarding the patient’s history of SGLT2 inhibitor use was available, and treatment was initiated as for typical DKA. However, 10 h after admission, blood glucose normalized and acidosis showed signs of improvement; insulin was tapered off, but the acidosis worsened. Although hyperchloraemia and lactic acidosis can cause high anion gap metabolic acidosis, in this case, the plasma chloride and lactate concentrations were normal, suggesting the presence of ketones and a diagnosis of DKA. It took 52 h to obtain improvement in acidosis with glucose supplementation up to 15 g/h, which is higher than the recommended dose of 7.5–12.5 g/h [12]. The definition of euglycaemic DKA based on the blood glucose concentration at admission was not met, but the hyperglycaemic state because of comorbid infections might have masked this condition. At the very least, the rapid decrease in blood glucose concentration after treatment suggests that the patient had a condition similar to DKA, which is associated with SGLT2 inhibitors. The lower blood glucose concentration and prolonged acidosis should have prompted earlier suspicion of SGLT2 inhibitor use. Glucose supplementation is necessary for patients with typical DKA, and critical when SGLT2 inhibitors are the cause of DKA.

Patients presenting to the emergency department often have insufficient medical information. The use of SGLT2 inhibitors should be suspected when a patient with DKA is only mildly hyperglycaemic and has prolonged acidosis.
